# Nuck Canal Endometriosis Following IVF Attempts in a Young Patient—Report of a Case

**DOI:** 10.3390/clinpract14040100

**Published:** 2024-06-28

**Authors:** Maria Papadoliopoulou, Ioannis Margaris, Athanasios Giannakis, Menelaos G. Samaras, Nikolaos V. Michalopoulos, Panayiotis Kokoropoulos, Ioannis Panayiotides, Nikolaos Arkadopoulos

**Affiliations:** 1 4th Department of Surgery, Attikon University Hospital, Medical School, National and Kapodistrian University of Athens, 1 Rimini Street, 12462 Athens, Greecepkokoropoulos@attikonhospital.gov.gr (P.K.);; 2 2nd Department of Radiology, Attikon University Hospital, Medical School, National and Kapodistrian University of Athens, 1 Rimini Street, 12462 Athens, Greece; 3 2nd Department of Pathology, Attikon University Hospital, Medical School, National and Kapodistrian University of Athens, 1 Rimini Street, 12462 Athens, Greece; msamara@attikonhospital.gov.gr (M.G.S.);

**Keywords:** endometriosis, canal of Nuck, inguinal cyst

## Abstract

Introduction: Endometriosis is a common benign condition affecting 10–15% of women of reproductive age. An unusual site of endometriosis is the canal of Nuck, which is a physiologically obliterated space in women spanning the area from the deep inguinal ring to the labia majora. Case presentation: A 37-year-old woman, with a past medical history of several in vitro fertilization attempts, presented with a right-sided painful inguinal mass. She was subsequently offered surgical exploration and excision of the lesion, which revealed the presence of endometrial glands and stroma. Discussion: Despite being a relatively common and benign pelvic condition, endometriosis can rarely manifest in the inguinal region, within the canal of Nuck. The treating physician should be cognizant of Nuck canal endometriosis, especially in young female patients presenting with an irreducible mass in the inguinal region and associated cyclic pain or infertility. Conclusion: When clinically and radiologically suspected, surgical excision is indicated to establish the diagnosis, provide symptomatic relief and guide further decision making.

## 1. Introduction

Endometriosis is a common benign condition affecting 10–15% of women of reproductive age [1]. It is characterized by the presence of endometrial glands and stroma in tissues outside the uterine cavity, most commonly in the pelvic parietal peritoneum. It manifests with a variety of symptoms, including pelvic pain, dyspareunia, dysuria, abundant irregular menstruation, diarrhea or constipation, infertility and chronic fatigue [1,2]. Localization of the disease outside the pelvis is uncommon; yet it has been described in other sites including the abdominal cavity, thoracic cavity and the central nervous system. A particularly unusual site of endometriosis is the canal of Nuck, which is a physiologically obliterated space in women spanning the area from the deep inguinal ring to the labia majora. Firstly, described by anatomist Anton Nuck, canal of Nuck is a female developmental abnormality, originating from an incompletely obliterated processus vaginalis [3]. It, therefore, represents a patent peritoneal space within the inguinal region, which may provide a route for superficial dissemination of endometriosis. We herein describe an interesting case of Nuck canal endometriosis that we treated in our department.

## 2. Case Presentation

A 37-year-old woman presented to the outpatient surgical clinic with a six-month history of an enlarging and painful right inguinal swelling, without further accompanying symptoms. She specifically denied any symptoms of nausea and vomiting, obstipation, or worsening of the pain with straining. Her past medical history was significant for several in vitro fertilization attempts and hyperprolactinemia, for which she was treated with cabergoline. She had regular menstrual cycles, without dysmenorrhea or dyspareunia. The patient was never smoker and denied any previous surgical operations. 

Physical examination revealed the presence of a well-defined round lump in her right inguinal region. The skin was normal appearing, without any signs of overlying erythema. On palpation, the mass was firm and irreducible, without local tenderness. No cough impulse was noted. The rest of the examination was unremarkable. Full blood count and biochemical values were within the normal limits.

**Figure 1:**
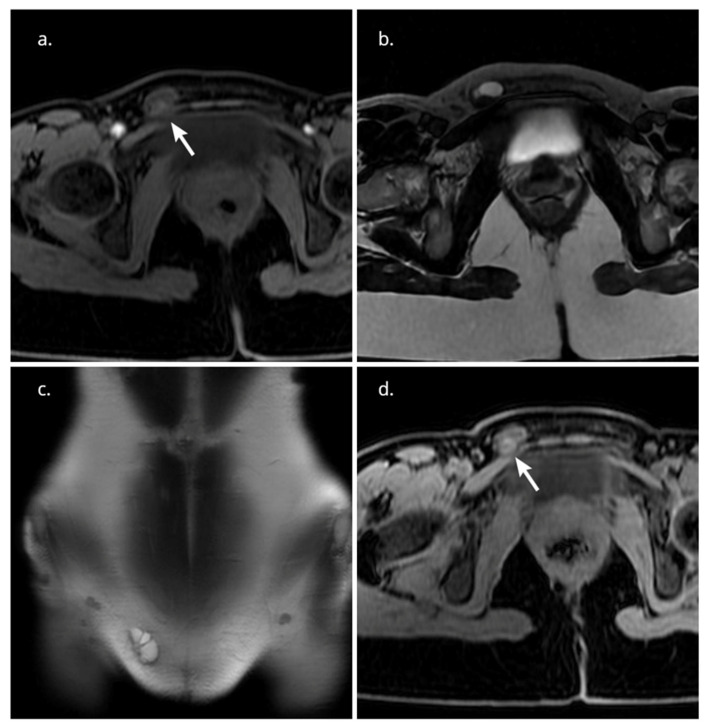
(**a**) Axial T1-weighted images with fat suppression: 2.8 cm hypointense lesion in the right inguinal canal with small hyperintense, probably hemorrhagic foci in the posterior part (white arrow). (**b**) Axial T2-weighted images: the lesion demonstrates hyperintense signal with hypointense foci in its posterior part. (**c**) Coronal T2-weighted images: hyperintense lobulated, septated cystic mass in the right inguinal canal. (**d**) Axial T1-weighted images with fat suppression after administration of gadolinium-based contrast material: Slightly enhancing foci in the posterior part of the lesion (white arrow).

Magnetic resonance imaging (MRI) revealed a lobulated, septated cystic mass in the right inguinal canal, which was hyperintense on T2-weighted images and mainly hypointense on T1-weighted images. The small hyperintense foci on T1-weighted images most probably indicated hemorrhagic areas. After intravenous gadolinium administration, the mass showed only slight enhancement (Figure 1).

**Figure 2:**
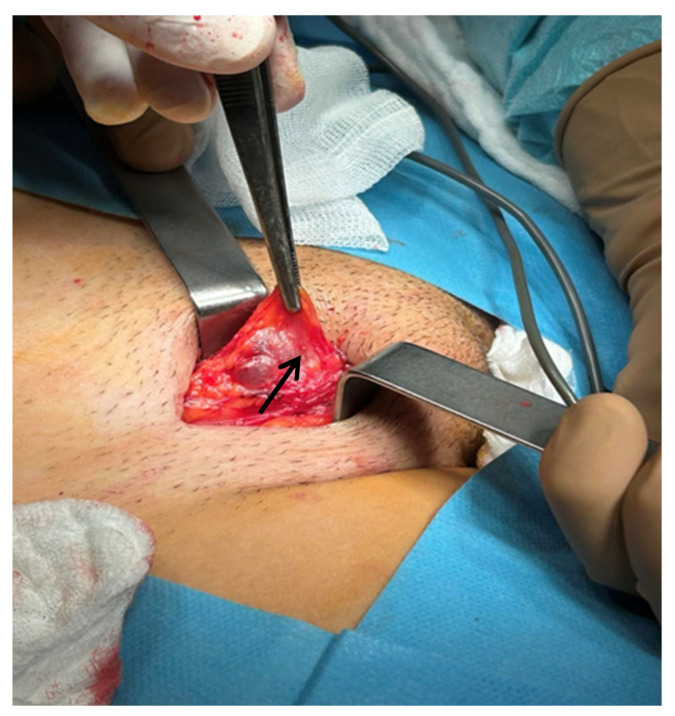
Intraoperatively, a cystic inguinal canal lesion was found, in close proximity to the round ligament of the uterus (blank arrow).

Subsequently, after discussing with the patient and obtaining informed consent, we proceeded with an elective inguinal exploration. During the operation, a cystic inguinal canal lesion was found, in close proximity to the round ligament of the uterus (Figure 2). The lesion was completely excised and sent for histological examination. The patient was discharged on the following day.

**Figure 3:**
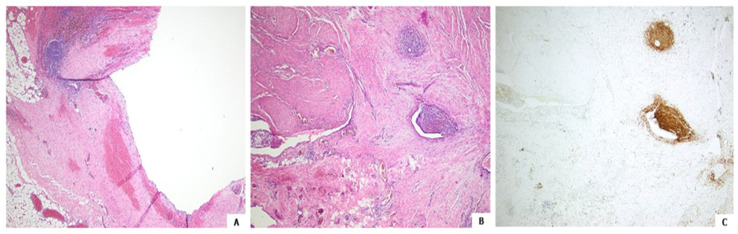
Endometriotic cyst. Attenuated epithelial lining, hemorrhage and pigmented histiocytes adjacent to endometrial stroma ((**A**), hematoxylin and eosin, 40×). Endometrial glands and stroma within the cystic wall ((**B**), hematoxylin and eosin, 40×). Endometrial stromal cells highlighted by CD10 immunoreactivity ((**C**), CD10 immunostain, 40×).

Histology after hematoxylin and eosin staining followed by immunochemistry showed the lesion to be an endometriosis focus, with both the cyst and adjacent glands focally lined with endometrial-like epithelium; CD10 immunopositive stromal cells were also seen (Figure 3) [4,5].

The patient was referred to a gynecologist for further assessment. She remained recurrence free and asymptomatic four months after the operation. 

## 3. Discussion

Endometriosis is a chronic estrogen-dependent clinical entity, associated with significant morbidity, primarily stemming from pain-related symptoms and possible infertility. The notion that retrograde menstruation through patent fallopian tubes can lead to implantation, survival and growth of endometrial cells outside the native organ has gained widespread acceptance [1]. However, recent evidence suggests a multifactorial pathogenesis, including intrinsic genetic and immunological defects [6]. 

With regard to location, pelvic endometriosis is the most prevalent [7]. Infrequently, the presence of endometriomas in the inguinal region or the Nuck canal has been reported. Following its first description by the 17th-century anatomist Anton Nuck, the namesake is a female developmental abnormality, originating from an incompletely obliterated processus vaginalis. It describes a potential space between the peritoneal cavity, the inguinal canal, and the labia majora [3]. Hypotheses supporting the presence of endometrial tissue within the canal of Nuck include the emergence from the round ligament, the metaplasia of the celomic epithelium or the above-mentioned retrograde menstruation theory [8].

We reported an interesting case of a unilateral painful groin lump in a young female patient, which was ultimately identified as a Nuck canal endometrioma. In a recent systematic review by Dalkalitsis et al., involving 133 patients with inguinal endometriosis, the mean age at diagnosis was 36 years and most of the patients presented with a right-sided inguinal mass, which correlates well with our report [8]. Even though the right-sided predilection is of unknown significance, it has been suggested that the presence of the sigmoid colon on the left side may exert a protective effect and inhibit retrograde menstruation through the ipsilateral canal of Nuck. An interesting fact is that approximately only half of the included patients reported a cyclic or catamenial pattern of pain, an otherwise predominant feature of endometriosis, which was also absent in our case. Furthermore, our reported patient had a significant medical history for multiple in vitro fertilization attempts, which could be indicative of infertility related to the underlying condition. 

In cases of unilateral painful inguinal enlargements, the differential diagnosis includes groin hernias, hydroceles, cysts, abscesses, vascular conditions or neoplasms [3,9]. Physical examination is of utmost importance and can reveal a magnitude of clinical sings. A growing bulge during strain, coughing, or when performing the Valsalva maneuver, can be indicative of the presence of an inguinal hernia or a hydrocele communicating with the peritoneal cavity. Clinical manifestations of inflammatory conditions may include fever, local redness, swelling and tenderness. A varicocele may appear with enlarging varicose veins during strain. Neoplastic lesions, even though rare and usually benign, can also originate from within the canal of Nuck and they can often be misdiagnosed as hernias. However, a right irreducible cystic mass in a female patient of reproductive age, especially when related to cyclic pain or a history suggestive of infertility, must turn the physician’s attention towards a potential endometriotic implantation. Further investigations are often warranted, in order to delineate the exact pathology and guide decision making. Ultrasonography might be a particularly useful initial investigation, since it is a readily available, practical, easy to perform and safe imaging modality. In cases of diagnostic uncertainty, computed tomography (CT), magnetic resonance imaging (MRI) or positron-emission tomography (PET) have reportedly been utilized [10].

Surgical excision is recommended as the treatment of choice for inguinal endometriosis [8,10,11]. Excision of the mass can establish a definite diagnosis and relieve from related symptoms, even though a minority of patients might experience a disease recurrence within months or years [8]. Furthermore, in rare cases, endometriosis within the canal of Nuck may give rise to malignant neoplasms, including clear cell adenocarcinoma, papillary adenocarcinoma and endometrial stromal sarcoma, necessitating additional staging or surgical procedures [10]. Hence, histological diagnosis is of utmost importance and should be pursued whenever possible. The presence of endometrial tissue glands and stroma are usually evident on histopathological examination of the extirpated lump or cyst. In controversial cases, staining for CD10 or mullerian epithelial tumor markers such as interferon-inducible transmembrane protein-1, and nuclear expression of PAX8 can be employed [4,5]. All patients should be referred to a gynecologist for further examination and assessment. Accordingly, an exploratory laparoscopy might be indicated, in order to identify and target additional intraabdominal sites of endometriosis. Finally, postoperative hormone suppression therapy can be administered, with recent evidence suggesting that it can significantly reduce the risk of endometriosis recurrence and postoperative pain [12].

Endometriosis is a highly underrecognized condition, mainly because of the presence of nonspecific and diverse symptoms and signs. Significant delays in the diagnosis of the disease have also been reported. However, the number of patients seeking care for infertility is growing and recent advances in assisted reproduction techniques have increased awareness of the disease among physicians and patients alike. A possible concern might be the effect of ovarian stimulation on endometriotic lesions during in vitro fertilization attempts, since it can possibly lead to exaggerated symptoms or lesion growth. In any case, we anticipate that in the future, endometriosis manifestations will likely become more evident and the disease more frequently recognized and diagnosed, even in atypical locations such as the canal of Nuck. Despite the fact that general surgeons may provide the initial treatment in such cases, endometriosis poses significant challenges and mandates a multidisciplinary treatment strategy to mange the full spectrum of the disease.

## 4. Conclusions

We presented a rare case of endometriosis within the canal of Nuck. The treating physician should be cognizant of the condition, especially in young female patients presenting with an irreducible mass in the inguinal region and associated cyclic pain or infertility. When clinically and radiologically suspected, surgical extirpation of the lesion is indicated if clinically feasible to establish the diagnosis, exclude malignant disease, provide symptomatic relief and guide further decision making. 

## Data Availability

The raw data supporting the conclusions of this article will be made available by the authors on request.
